# A Highly Sensitive CMOS Digital Hall Sensor for Low Magnetic Field Applications

**DOI:** 10.3390/s120202162

**Published:** 2012-02-15

**Authors:** Yue Xu, Hong-Bin Pan, Shu-Zhuan He, Li Li

**Affiliations:** 1 School of Electronic Science & Engineering, Nanjing University, Nanjing 210093, China; E-Mails: heshuzhuan@gmail.com (S.-Z.H.); lili@nju.edu.cn (L.L.); 2 College of Electronic Science & Engineering, Nanjing University of Posts and Telecommunications, Nanjing 210003, China; E-Mail: yuex@njupt.edu.cn

**Keywords:** Hall sensor, CMOS technology, dynamic offset cancellation, chopped technique

## Abstract

Integrated CMOS Hall sensors have been widely used to measure magnetic fields. However, they are difficult to work with in a low magnetic field environment due to their low sensitivity and large offset. This paper describes a highly sensitive digital Hall sensor fabricated in 0.18 μm high voltage CMOS technology for low field applications. The sensor consists of a switched cross-shaped Hall plate and a novel signal conditioner. It effectively eliminates offset and low frequency 1/f noise by applying a dynamic quadrature offset cancellation technique. The measured results show the optimal Hall plate achieves a high current related sensitivity of about 310 V/AT. The whole sensor has a remarkable ability to measure a minimum ±2 mT magnetic field and output a digital Hall signal in a wide temperature range from −40 °C to 120 °C.

## 1. Introduction

Presently, Hall magnetic field sensors are widely established for their great applications in industrial control systems, intelligent instruments, and consumer electronic products, *etc*. They are used not only for direct measurement of magnetic fields, but also for non-direct measurements, like speed or position, *etc*. Hall devices can be realized in standard integrated circuit processes such as the bipolar or CMOS technologies. Compared to bipolar Hall devices, CMOS Hall devices exhibit the following advantages: high reliability, small size, low cost and compatibility with other CMOS technologies [1–4]. Unfortunately, integrated CMOS Hall sensors also suffer from a lot of non-idealities [2–4]. First of all, their magnetic field sensitivity is very low. For instance, in a linear or angular position Hall sensor, the value of magnetic field is usually 5 mT at one cm distance from a magnet which has a magnetic field of around 0.1 T. Under this magnetic field, the CMOS Hall device gives a weak output signal of hundreds of micro-volts. Second, its offset is rather high. A CMOS Hall device is very vulnerable to process fluctuation, temperature drift and package-induced stress. These negative factors induce serious offset voltage and low frequency 1/f noise which may be large enough to obscure the Hall signal. In addition, CMOS operational amplifiers (OP-AMPs) used for Hall signal conditioning have poor performance in terms of offset and 1/f noise compared to bipolar OP-AMPs. For example, the typical value of the offset of a CMOS OP-AMP is as large as ±2 mV [5].

Therefore, the low magnetic sensitivity and the large offset of Hall sensors limit both the minimum value of the magnetic field that can be measured as well as the accuracy of the measurements. So far the techniques used to reduce the offset and 1/f noise from the electronic circuits, can be mainly divided into auto-zero (AZ), correlated double sampling (CDS) and chopper stabilization (CHS) techniques [6]. Compared with the AZ and CDS, the CHS technique can effectively eliminate the offset and 1/f noise of the electronics without requiring any low-pass filtering [7–9]. It transposes the signal to a higher frequency, where there is no low frequency 1/f noise, and then demodulates it back to the baseband after amplification. However, the CHS technique only effectively removes the offset and 1/f noise of the amplifiers, but it cannot satisfactorily suppress the external input offset and 1/f noise. In order to eliminate these non-ideal factors simultaneously, a quasi-chopped technique for dynamic offset cancellation, *i.e.*, the so-called spinning current technique, has been widely employed in Hall sensors [9,10].

This paper deals with a highly sensitive digital Hall sensor fabricated in 0.18 μm high voltage (HV) CMOS technology for low magnetic field applications. A new chopper stabilized instrumental chain is employed to perform the dynamic offset cancellation, which mainly consists of an optimal switched Hall plate, and a novel and simple signal conditioner. In particular, the proposed signal conditioner features a switched hysteresis comparator to replace the sample-hold circuit and Schmitt trigger of conventional signal conditioner, which further reduces the size and cost of the proposed Hall sensor.

This paper is arranged as follows: first, the design and optimization of the CMOS horizontal Hall plate is briefly introduced. Further, the structure of an analog front-end with the dynamic offset cancellation is described in detail. Then, the simulation and experimental results are presented and discussed. Finally, conclusions are drawn.

## 2. Cross-Shaped Hall Plate

**Figure 1.**
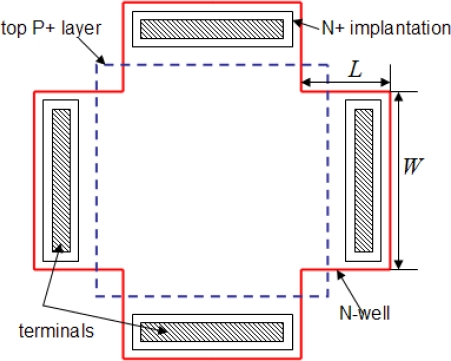
Top view of a conventional cross-shape Hall plate.

The cross-shaped Hall plate as a horizontal Hall device has been broadly used due to its relatively high sensitivity and low offset. The structure of the CMOS cross-shaped Hall plate is schematically shown in Figure 1. It is fabricated in an N-well diffusion area which is built in a P-type substrate, with four N+ doped terminals [11–13]. The 90° rotation symmetrical structure makes it well suitable for spinning current use where the biasing and sensing terminals are periodically permutated. In order to reduce the 1/f noise and carrier surface losses, a shallow P+ top layer often covers the surface of the N-well. The P+ top layer and P-type substrate are usually connected to ground. When a voltage *V* or current *I* bias is supplied via one pair of terminals and a perpendicular magnetic field *B_Z_* is applied to the device surface, the Hall voltage *V_H_* appears on the other pair of terminals due to the Hall effect. Considering the geometry of a real Hall plate, *V_H_* can be expressed with the current related sensitivity *S_I_* [13]:

(1) $$V_{H}=S_{I}\;{\mathrm{IB}}_{Z},$$

with $S_{I}=G\mu_{H}\;R_{\mathrm{square}}=\frac{G\cdot r_{H}}{{\mathrm{qn}}_{\mathrm{NW}}\;t_{\mathrm{NW}}}$. *S_I_* is determined by the geometrical correction factor *G*, the Hall mobility *μ_H_* or the Hall factor *r_H_*, the doping concentration *n_NW_* of the N-well, and the effective depth of the N-well *t_NW_*.

Equation (1) can be rewritten with the voltage related sensitivity *S_V_*:

(2) $$V_{H}=S_{V}\;{\mathrm{VB}}_{Z},$$

with $S_{V}=\frac{S_{I}}{R_{\mathrm{square}}N_{\mathrm{square}}}=\frac{G}{2\frac{L}{W}+\frac{2}{3}}\;\mu_{H}$, *L* and *W* are the finger length and finger width of the cross-shaped Hall plate, respectively.

For a cross-shaped Hall plate, the geometrical correction factor can be calculated by [13]:

(3) $$G=1-5.0267\frac{\theta_{H}}{\text{tan}(\theta_{H})}e^{-\frac{\pi }{2}\frac{W+L}{W}}$$

where *θ_n_* is the Hall angle, equal to tan^−1^(*μ_H_B*).

Equation (1) means that *S_I_* is inversely proportional to the carrier concentration of the N-well. Therefore, a Hall device fabricated by a standard CMOS process has a low sensitivity due to high N-well doping concentration. In order to improve the current related sensitivity, we select a HV CMOS process to fabricate the Hall plate as it can provide an obviously lower N-well doping level than the standard CMOS process, despite a relatively deep N-well depth. On the other hand, the geometrical correction factor should also be enhanced, which is determined by the ratio of finger length *L* to finger width *W* in terms of Equation (3). In order to improve the voltage related sensitivity without reducing the current related sensitivity too much, an optimal cross geometry (*W/L* = 2) has been reasonably selected in the layout design.

It is well known that CMOS Hall devices seriously suffer from a large offset. One of the main origins of offset comes from the mask-misalignment, which can be minimized by designing a Hall device with an appropriate and symmetric layout. In fact, the masks defining the terminals and N-well implant active layer of the Hall plate could be shifted or rotated relative to each other during photolithography. Any misalignment between terminals mask and the N-well mask will result in an offset, even in the absence of magnetic field. However, the smaller terminals designed within the N-well could lead to a larger masks misalignment. In the layout design, an optimized cross-shaped Hall plate structure is developed. Compared to the conventional Hall plate, the length of terminals reaches a maximum allowable value in the N-well for a given technology. Thus, the effect of the masks misalignment on the offset can be greatly reduced.

## 3. Front-End Signal Conditioning

**Figure 2.**
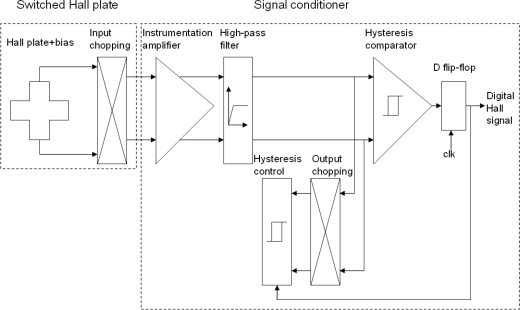
Block diagram of the new chopper stabilized instrumental chain.

The block diagram of the new chopper stabilized instrumental chain is illustrated in Figure 2. At first, applying the spinning current technique, the output and supply terminals of Hall plate are periodically interchanged so that the useful Hall signals are separated from the offset and 1/f noise through input chopping modulation. Then, the modulated signals are amplified by a differential instrumentation amplifier. After this amplification, two high-pass filters remove the unwanted offset and 1/f noise. Finally, the output signal passing through the filters is demodulated and the digital Hall signal is generated by a switched hysteresis comparator.

### 3.1. Switched Hall plate

**Figure 3.**
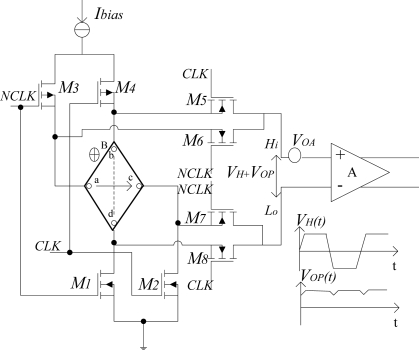
Switched Hall plate.

Figure 3 shows the switched Hall plate in Figure 2. Since the 90° rotation symmetrical Hall plate can be considered as a distributed resistive Wheatstone bridge from a dc point of view, the dynamic offset cancellation can be achieved by the spinning current method [3,4]. By periodical supply and output terminals permutation, the quadrature states are generated. One pair of complementary clocks of 100 kHz produce 0° and 90° states respectively. When CLK is high level, M2, M3, M5, and M8 turn on. The terminal *a* and terminal *c* of the Hall plate are connected to power and ground. Then current flows from terminal *a* to terminal *c*, and Hall signal appears between terminal *b* and terminal *d*. When NCLK is high level, M1, M4, M6 and M7 turn on, so there is a current flowing from terminal *b* to terminal *d*. Accordingly, a Hall signal is present between terminal *c* and terminal *a*. Thus, if the change of the magnetic field is much slower than the clock frequency, the differential output Hall voltage *V_H_* periodically changes its polarities with the same magnitude in the course of current spinning. On the contrary, the differential output offset voltage *V_OP_* always keeps the same magnitude and a constant polarity, as the same imbalance occurs in adjacent branches of the equivalent Wheatstone bridge network. It is important to note that the offset *V_OA_* of the instrumentation amplifier becomes indistinguishable from *V_OP_*. Consequently, a demodulation should be performed to extract the Hall signal and eliminate the Hall offset and the instrumentation amplifier’s offset simultaneously by the following signal conditioner at no extra cost.

### 3.2. Signal Conditioner

The traditional signal conditioners execute sample-and-hold (S/H) and adding functions to remove offset without using low-pass filters [3,8]. First, the two differential outputs of the instrumentation amplifier are sampled and hold by S/H circuits during 0° and 90° states respectively. Next, the outputs of S/H circuits input the summing OP-AMP. Finally, the offset can be cancelled out by the summing OP-AMP. However, this signal conditioner layout requires four completely differential S/H circuits and a summing OP-AMP, thus it requires too large a chip size to fabricate four S/H capacitances. Moreover, the circuit structure is much more complicated. Later, a simplified circuit configuration was proposed [14]. Here, a capacitance clocked by sampling clock is used to realize the adding function, taking place of a summing OP-AMP. Further, it only needs two S/H capacitances, and the total number of capacitances decreases from four to three. Nevertheless, this circuit requires four-phase different clocks, and the timing relationship in the circuit is much more complex.

**Figure 4.**
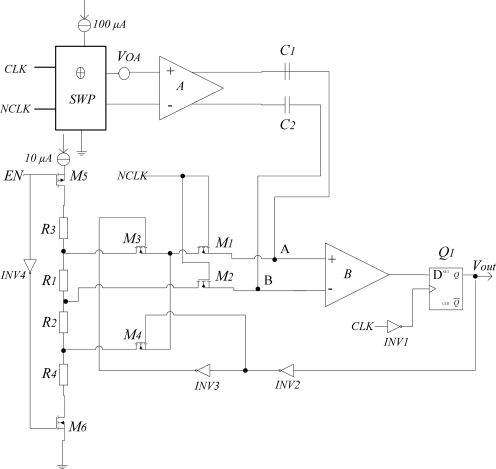
Signal conditioner of the digital Hall sensor.

In this work, we propose a signal conditioner based on a high-pass filtering—demodulation configuration, as shown in Figure 4. Here, the switched Hall plate is represented by block SWP. EN is the enable signal and high level is effective. Compared to other similar signal conditioners [15,16], the proposed signal conditioner has a simpler structure. In addition to the instrumentation amplifier A, it only consists of two high-pass filters and a switched hysteresis comparator B. The circuit properly works as follows: during the 0° state, the differential input voltage of the instrumentation amplifier is:

(4) $$V_{i}(0^{{}^{\circ}})=V_{H}+\left|V_{\mathrm{OP}}(0^{{}^{\circ}})\right|+\left|V_{\mathrm{OA}}\right|.$$

During the 90° state, the differential input voltage of the instrumentation amplifier changes to:

(5) $$V_{i}(90^{{}^{\circ}})=-V_{H}+\left|V_{\mathrm{OP}}(90^{{}^{\circ}})\right|+\left|V_{\mathrm{OA}}\right|.$$

After it is amplified by the instrumentation amplifier, the Hall signal can pass through the high-pass filters, but the offset and 1/f noise are blocked. It is important to notice that a simple passive first order high-pass filter is sufficient to perfectly cancel the offset and 1/f noise. The cut-off frequency of the high-pass filter has to be higher than the 1/f noise frequency, which is typically between 500 Hz and 1 kHz, so a first order high-pass filter with 2 kHz cut-off frequency can achieve this requirement. The cut-off frequency is determined by 1/C_1_R_AB_ or 1/C_2_R_AB_, where R_AB_ is the equivalent resistance between the points A and B. In order to obtain low 1/f noise performance, C_1_ and C_2_ are set to 10 pF.

When NCLK is high level, switches M1 and M2 turn on, then, the differential input voltage of the comparator B is clamped to:

(6) $$V_{\mathrm{AB}}=V_{\mathrm{th}}$$

where *V_th_* is a trigger threshold level and its polarity is controlled by the feedback Hall output signal. When the Hall output signal is high level, switch M3 turns on and *V_th_* is equal to *V_R1_* (the current of 10 μA flowing across resistor *R_1_*). Otherwise, switch M4 turns on, then *V_th_* becomes −*V_R2_* (the current of 10 μA flowing across resistor *R_2_*). We selected *R_1_* = *R_2_* = *R_3_* = *R_4_* = 2 K to make *V_th_* = ±20 mV. At the moment, the cut-off frequency of the high-pass filters is much higher than the chopping frequency of 100 KHz, hence both Hall signal and offset and 1/f noise are blocked.

When NCLK is low level, switches M1 and M2 turn off. At this time, the cut-off frequency of the high-pass filters becomes less than 100 kHz but higher than 2 kHz. Thus, only the Hall signal can pass through the high-pass filters. Since the amount of electric charge on the capacitances C1 and C2 remains unchanged, the differential input voltage of the comparator B changes to:

(7) $$V_{\mathrm{AB}}=2A_{u}\;V_{H}+V_{\mathrm{th}}$$

where, *A_u_* is the voltage gain of the instrumentation amplifier A, and the residual offset voltage is neglected.

First assume that the initial state is *V_A_* > *V_B_*, so the output voltage of comparator B is a high level. At this time, *V_th_* is equal to *V_R1_*. When 2*A_u_V_H_* reversely increases more than |*V_R1_*|, the comparator B outputs a low level and *V_th_* becomes −*V_R2_*. Only when the value of 2*A_u_V_H_* forward increases more than |*V_R2_*|, the output becomes a high level. In order to output a standard CMOS level, a D flip-flop (DFF) is used.

Therefore, the proposed signal conditioner not only effectively eliminates the offset and 1/f noise, but also realizes the hysteresis characteristics of a digital Hall sensor without a Schmitt trigger. Meanwhile, the whole signal conditioner only needs two capacitances, which further make the chip cost-effective.

## 4. Circuit Simulations

**Figure 5.**
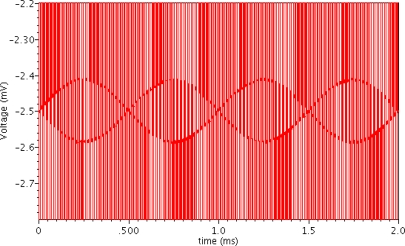
Simulated transient voltage waveform between the differential inputs of the instrumentation amplifier.

**Figure 6.**
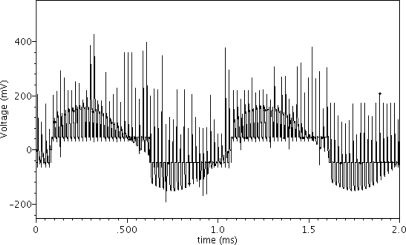
Simulated transient voltage waveform between the differential inputs of the comparator.

**Figure 7.**
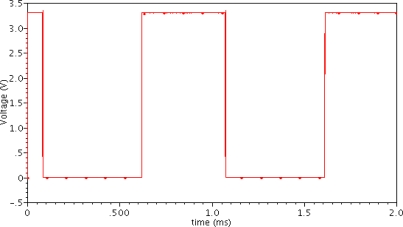
Simulated digital Hall output signal of the signal conditioner.

A SPICE simulation of the front-end chopper stabilized instrumental chain was performed with 100 kHz chopping clock frequency using a Cadence spectre simulator. The simulation model parameters of the devices were derived from the X-FAB 0.18 μm HV CMOS technology. The Hall plate is modeled by an equivalent simulation model written in Verilog-A language [13]. The Hall plate model produces a 1 kHz sinusoidal Hall output signal of 80 μV and a dc output offset of 2.5 mV when the input bias current is 100 μA and the perpendicular magnetic field is 2.5 mT. After the Hall plate output signals are modulated at 100 kHz by applying the spinning current technique, they are fed into the instrumentation amplifier for amplification. Figure 5 illustrates the transient voltage waveform between the differential inputs of the instrumentation amplifier. Unfortunately, some parasitic spikes are obviously observed during commutations. These spikes are generated by the various non-idealities of the switches, including charge injection, clock feed-through and parasitic capacitances of Hall plate and switches. Although a dummy switch can reduce the charge injection, it will increase the complexity of the spinning current circuit. Since the RC time of the spikes is dominated by the resistance of the Hall device and the parasitic capacitances, the best method to suppress these spikes is to reduce the parasitic capacitances. Therefore, we properly reduce the size of the switches without increasing the on-resistance too much and we employ a small Hall plate to reduce the parasitic capacitances in our design. Figure 6 shows the transient simulation voltage waveform at the differential inputs of the comparator B. It is clearly observed that the high-frequency Hall signal is demodulated into the original low-frequency signal and the offset is effectively eliminated by the high-pass filters. The digital Hall output signal waveform is shown in Figure 7. It can be seen that when the input Hall signal changes polarity, that is the magnetic field changes direction, the DFF output level changes synchronously. The simulation results also show that when the amplitude of the input Hall signal increases to 0.15 mV the signal conditioner can even cancel a maximum input Hall offset of 10 mV, which means that the signal conditioner can tolerate a larger offset if the Hall signal becomes larger. The simulated results indicate the improved signal conditioner has a remarkable ability to suppress the large offset and amplify the weak Hall signal.

## 5. Experimental Results

**Figure 8.**
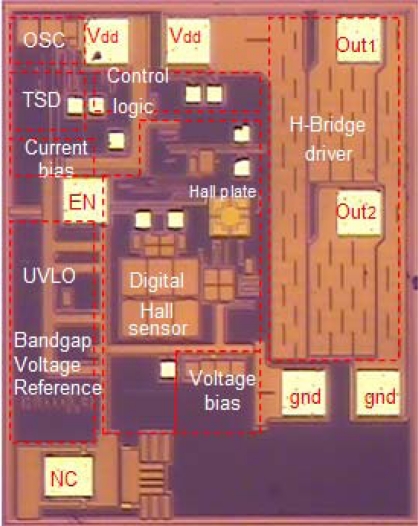
Microphotograph of the digital Hall sensor die.

A highly sensitive digital Hall sensor using the dynamic offset cancellation technique was implemented in a monolithic brushless DC motor driver chip based on the X-FAB 0.18 μm HV CMOS process for detecting magnetic pole position. A microphotograph of the driver chip is shown in Figure 8. The cross-shaped Hall plate measuring 64 × 64 μm^2^ is situated in the central region of the chip. The spinning current circuit and the signal conditioner are located close to the Hall device. In addition, the single-chip motor driver also integrates other important functional blocks such as control logic, H-bridge driver, oscillator, bandgap voltage reference, thermal shutdown (TSD) and under voltage lockout (UVLO) protection, *etc*.

Firstly, the performances of the single Hall device were tested by using a special Hall effect measuring instrument NDWH-648. The instrument can achieve a Hall voltage resolution of 1 μV, providing a sufficiently high measuring accuracy over the measuring temperature range from 77 K to 400 K. When the Hall plate is biased with 100 μA DC current, the current related sensitivity was measured at room temperature. A comparison of the current related sensitivity has been made between the proposed Hall device and the reported CMOS Hall devices, as shown in Table 1.

**Figure 9.**
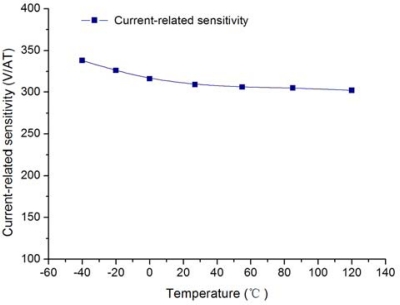
Measurement of the current-related sensitivity versus temperature.

It is obvious that a higher sensitivity of about 310 V/AT is achieved in this work. Figure 9 shows the thermal drift of the current related sensitivity within an industrial temperature range of −40 °C–120 °C.

**Figure 10.**
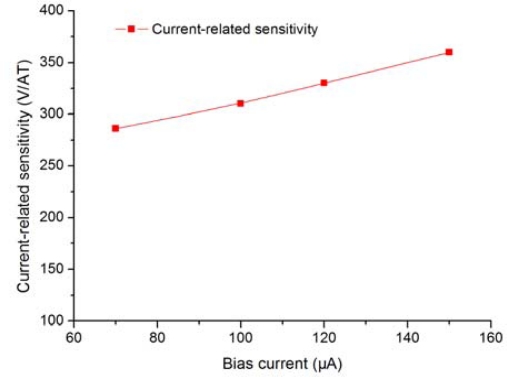
Measurement of the variation of the current-related sensitivity with the biasing current.

The temperature first order coefficient of sensitivity is estimated to about 800 ppm/K. The variation of the current related sensitivity with the DC bias current is shown in Figure 10. Because of the junction effect increasing with the bias current, the effective thickness of the active area of the Hall plate is reduced. Thus, we observe that the sensitivity is increased with the bias current. The static Hall offset voltage between one pair of terminals was measured when the substrate is grounded and two other terminals are applied to 1.0 V and 0 V. A small Hall offset about 1 mV is obtained at temperature = 27 °C.

**Figure 11.**
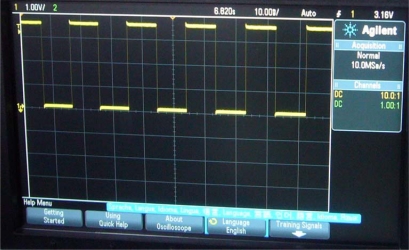
Digital output of the Hall sensor displayed on an Agilent 3032A oscilloscope.

Next, the functions of the whole Hall sensor were measured. The digital output of the Hall sensor was observed by using an Agilent 3032A oscilloscope, as illustrated in Figure 11. It can be seen that the output level of the Hall sensor changes synchronously when the magnetic field changes from −2 mT to 2 mT. A minimum detectable magnetic field of ±2 mT is obtained, showing a hysteretic characteristic of 4 mT. Note that the detectable sensitivity could be further improved by reducing the threshold voltage of the hysteresis comparator, whereas a too low threshold voltage will cause a poor anti-jamming ability of the hysteresis comparator. Therefore, the minimum detection sensitivity of ±2 mT is difficult to improve. In addition, the test results show that the Hall sensor can work well as the supply voltage changes from 2 V to 4 V while the temperature ranges from −40 °C to 120 °C.

Table 2 summarizes the measured important parameters of the digital Hall sensor die, which suggests that the CMOS integrated digital Hall sensor can provide high sensitivity and better temperature stability over a wide temperature range.

## 6. Conclusions

A highly sensitive digital Hall magnetic sensor using the X-FAB 0.18 μm HV CMOS technology is introduced. The cross-shaped structure of Hall device is optimized to reduce Hall offset and improve sensitivity. In order to eliminate the relatively large offset, including Hall offset, amplifier’s offset and 1/f noise, the dynamic offset cancellation technique through Hall current spinning is applied. A novel signal conditioner with a simple structure is proposed for saving chip area and improving the performance of the sensor. The recovery of digital Hall output and offset cancellation are achieved with only two high-pass filters and a switch-controlled comparator. The whole signal conditioner only requires a pair of complementary clocks. Additionally, it is convenient to change the hysteresis characteristics by adjusting resistances, without needing an actual Schmitt trigger. The experimental results show that the sensor has a remarkable ability to measure a minimum ±2 mT magnetic field and output a digital Hall signal over a wide temperature range from −40 °C to 120 °C. Therefore, this Hall sensor is well suited for low magnetic field applications, such as integrated brushless DC motor drivers which require small chip size and high sensitivity.

## Figures and Tables

**Table 1.** Comparison of the current related sensitivity between the proposed single Hall device and the reported Hall devices.

	**Current Related Sensitivity**	**Technology**
This work	310 V/AT	0.18 μm HV CMOS
[7]	90 V/AT	0.35 μm CMOS
[16]	95 V/AT	0.35 μm CMOS
[17]	180 V/AT	0.8 μm CMOS

**Table 2.** Typical characteristics of Hall sensor.

**Parameters**	**Value**
Supply voltage	2–4 V
Hall plate sensitivity @100μA	310 V/AT
Original Hall plate offset @1V	1 mV
Operating point B_OP_ @27°C	2 mT
Release point B_OP_ @27°C	−2 mT
Hysteresis @27°C	4 mT
Operating temperature range	−40–120 °C

## References

[1] Bellekom S. (1999). CMOS *versus* bipolar Hall plates regarding offset correction. Sens. Actuat. A.

[2] Popovic R.S., Randjelovic Z., Manic D. (2001). Integrated Hall-effect magnetic sensors. Sens. Actuat. A.

[3] Randjelovic Z.B., Kayal M., Popovic R., Blanchard H. (2002). High sensitive Hall magnetic sensor Microsystem in CMOS technology. IEEE J. Solid-St. Circ.

[4] Blanchard H., De M.F., Hu B.J., Popovic R.S. (2000). Highly sensitive Hall sensor in CMOS technology. Sens. Actuat. A.

[5] Bakker A., Thiele K., Hui J.J. (2000). A CMOS nested-chopper instrumental amplifier with 100-nV offset. IEEE J. Solid-St. Circ.

[6] Enz C.C., Temes G.C. (1996). Circuit techniques for reducing the effects of op-amp imperfections: Autozeroing correlated double sampling, and chopper stabilization. Proc. IEEE.

[7] Yang W.R., Ran F. CMOS Chopped Amplifier for Monolithic Magnetic Hall Sensor.

[8] Bilotti A., Monreal G. (1999). Chopper-stabilized amplifiers with a track-and-hold signal demodulator. IEEE Trans. Circ. Syst.-I.

[9] Frick V., Pascal J., Blondé J.-P., Hébrard L. Chopper Stabilized CMOS Integrated Front-End for Magnetic Field Measurement.

[10] Bilotti A., Monreal G., Vig R. (1997). Monolithic magnetic Hall sensor using dynamic quadrature offset cancellation. IEEE J. Solid-St. Circ.

[11] Jovanovic E., Pesic T., Pantic D. 3D Simulation of Cross-Shaped Hall Sensor and Its Equivalent Circuit Model.

[12] Schweda J., Riedling K. A Nonlinear Simulation Model for Integrated Hall Devices in CMOS Silicon Technology.

[13] Xu Y., Pan H.B. (2011). An improved equivalent simulation model for CMOS integrated Hall plates. Sensors.

[14] Hu Y., Yang W.R. CMOS Hall Sensor Using Dynamic Quadrature Offset Cancellation.

[15] Frick V., Hebrard L., Poure P., Braun F. CMOS Microsystem Front-End for MicroTesla Resolution Magnetic Field Measurement.

[16] Ouffoue C., Frick V., Kern C., Hébrard L. New Fully Differential Instrumental Chain for Hall Sensor Signal Conditioning Integrated in Standard 0.35 μm CMOS Process.

[17] Demierre M., Pesenti S., Frounchi J., Besse P.-A. (2002). Reference magnetic actuator for self-calibration of a very small Hall sensor array. Sens. Actuat. A.

